# Using the modified Delphi method to research the influencing factors of long-term health-related quality of life in patients with unruptured intracranial aneurysms after endovascular treatment

**DOI:** 10.1186/s41016-020-00186-1

**Published:** 2020-03-20

**Authors:** Xiao-Dong Zhai, Chun-Xiu Wang, Yong-Jie Ma, Jia-Xing Yu, Si-Shi Xiang, Han-Yi Jiao, Peng Shao, Xin Guan, Jun Wang, Hong-Qi Zhang

**Affiliations:** 1 Department of Neurosurgery, Xuanwu Hospital, Capital Medical University, No. 45 Changchun Street, Xicheng District, Beijing, 100053 China; 2 China International Neuroscience Institute (China-INI), Beijing, China; 3 Department of Evidence-Based Medicine, Xuanwu Hospital, Capital Medical University, Beijing, China

**Keywords:** Modified Delphi method, Unruptured intracranial aneurysms, Health-related quality of life

## Abstract

**Background:**

The purpose of this study was to use the modified Delphi method to identify the influencing factors of health-related quality of life (HRQoL) in patients with unruptured intracranial aneurysms (UIAs) after endovascular treatment.

**Methods:**

A modified Delphi method to obtain expert consensus on the content of potential influencing factors of HRQoL in patients with UIAs treated by endovascular intervention was employed. The research team consists of three neuroradiologists and one epidemiologist from Xuanwu Hospital of Capital Medical University. They randomly selected 21 well-known experts in cerebrovascular disease diagnosis and treatment as participating experts. The importance of the indicator is based on the 5-Likert scale. The standard deviation (SD), coefficient of variation (CV), mean ($\overline{x}$), and minimum and maximum scores of each indicator were calculated. The consistency was described by Kendall coefficient of concordance with a *p* value < 0.05 indicating that the expert consistency was high.

**Result:**

Twenty-one and 18 questionnaires were responded in 2 rounds, with effective response rates of 85.7% and 100.0%, respectively. The average authoritative coefficient (Cr) of all 21 experts was 0.88, familiarity with the indicators (Cs) was 0.82, and the judgment basis of the indicators (Ca) was 0.94. Eventually, the $\overline{x}$ values of arterial puncture hematoma, hyperlipidemia, gender, marital status, and hospitalization for other diseases were lower than 3.5; CV for marital status and gender was higher than 0.35. The Kendall coefficient of concordance in the first round was 0.19 (*p* < 0.001), and the second round was 0.15 (*p* < 0.001).

**Conclusion:**

In this study, the factors affecting the recovery of HRQoL after endovascular treatment in patients with UIAs were analyzed by the modified Delphi method, which provided a valuable evidence for the clinical management and daily life guidance for UIAs patients.

## Background

Advances and increasing availability of high-resolution imaging technologies have led to a higher rate of incidentally detected unruptured intracranial aneurysms (UIAs) [1, 2]. UIAs are a major public health problem and cause subarachnoid hemorrhage (SAH), intracerebral hemorrhage (ICH), or intraventricular hemorrhage (IVH) when they rupture. The prevalence of UIAs was approximately 7.0% in Chinese adults aged 35 to 75 years [2]. Health-related quality of life (HRQoL) is regarded as an objective evaluation of physical health, mental health, and social life for patients and is an important component of disease prognosis evaluation methods [3–5]. Previous studies reported that UIA patients who underwent endovascular treatment presented with significant lower HRQoL level than the general population with unknown cause [3, 4, 6]. Therefore, the purpose of this study was to use the modified Delphi method to identify the influencing factors of HRQoL in patients with UIAs after endovascular treatment.

## Methods

### Modified Delphi method

The modified Delphi method can help to fill the knowledge gap by reaching consensus using the knowledge and personal opinions of experts [7, 8]. The modified Delphi method typically presents participants with 2–4 rounds of a fixed set of questions. After each round, participants receive a summary of responses from the previous round. Based on this summary, participants may adjust their answers in the following round. This process continues until participants reach consensus or if no additional consensus is expected [9].

### The expert panel

The research team consists of three neuroradiologists and one epidemiologist from Xuanwu Hospital of Capital Medical University. Participants in modified Delphi method are usually experts on the topic on which the consensus is sought. Their scientific and practical understanding of potential factors and their experience and opinion on whether a factor is major or minor are crucial for Delphi method. There is a general recommendation of 15 to 30 participating experts for a modified Delphi method survey [8]. In addition, geographical spread was also taken into an account to enhance the representativeness of experts. As shown in Table 1, the research team randomly selected 21 experts in cerebrovascular diseases diagnosis and treatment from the national project 2016YFC1300800 as participating experts in the modified Delphi method.

**Table 1:** Basic information and authoritative evaluation of 18 experts

Expert ID	Gender	Age (years)	Work years	Degree	Title	Ca	Cs	Cr
1	Male	48	25	Doctor	Chief physician	0.9	0.9	0.9
2	Male	46	21	Doctor	Chief physician	1	0.9	0.95
3	Male	56	30	Doctor	Chief physician	1	0.9	0.95
4	Male	50	26	Doctor	Chief physician	1	0.9	0.95
5	Male	47	20	Doctor	Chief physician	0.8	0.7	0.75
6	Male	49	26	Doctor	Chief physician	0.9	0.9	0.9
7	Male	46	22	Doctor	Chief physician	0.9	0.9	0.9
8	Male	59	35	Bachelor	Chief physician	1	0.9	0.95
9	Male	55	32	Bachelor	Chief physician	0.9	0.7	0.8
10	Male	42	18	Doctor	Associate chief physician	0.9	0.7	0.8
11	Male	41	16	Doctor	Associate chief physician	0.8	0.9	0.85
12	Male	40	10	Doctor	Associate chief physician	1	0.9	0.95
13	Male	37	9	Doctor	Associate chief physician	1	0.7	0.85
14	Male	40	9	Doctor	Associate chief physician	1	0.9	0.95
15	Male	47	22	Doctor	Associate chief physician	0.9	0.9	0.9
16	Female	50	28	Master	Chief nurse	1	0.7	0.85
17	Female	38	18	Bachelor	Supervisor nurse	1	0.7	0.85
18	Female	46	27	Bachelor	Supervisor nurse	0.9	0.7	0.8
Mean						0.94	0.82	0.88

The research team developed the items for the first round of the modified Delphi method based on literature review and expert interview. Combined with the results of the first round of questionnaires and expert opinions, the results after statistical analysis of relevant items are sent to the experts again for a second round of review. The importance of the factors is based on the 5-Likert scale assignment: very important (5 points), important (4 points), normal (3 points), not important (2 points), and very unimportant (1 point). The familiarity of experts with the question is assigned from high to low (0.9 to 0.0), respectively.

### Statistical methods

Statistical analysis was performed using SPSS Statistics version 24.0 software (IBM Corp., Armonk, New York, USA). The mean ($\overline{x}$), standard deviation (SD), coefficient of variation (CV), and minimum and maximum scores of each indicator are calculated and counted. CV demonstrates the degree of relative concentration among the experts. The Kendall coefficient of concordance was used to describe the degree of consistency of the expert opinions, and the consistency was higher at *p* < 0.05.

## Results

### Active coefficient of the experts

The active coefficient of the experts is usually expressed by the response rate of the questionnaire, indicating the concern of the experts on the research of the project. The response rate of the questionnaire which exceeds 70% is regarded as a higher active coefficient. The study received 21 and 18 questionnaires, respectively, with effective response rates of 85.7% and 100%, respectively.

### Authority coefficient of the experts

The authoritative coefficient (Cr) is usually determined by two factors: the judgment basis of the indicators (Ca) and the familiarity with the indicator (Cs). The calculation formula is Cr = (Ca + Cs)/2. The value of Ca and Cs was obtained mainly through self-evaluation of the experts. The larger the Cr value, the higher the authoritative coefficient, and CR ≥ 0.7 is regarded as the high degree of authority coefficient. The average Ca of all experts was 0.94 (Table 1), and the average Cs was 0.82. Therefore, the average Cr of all experts was 0.88, indicating that the experts included in this study obtained a high degree of authority.

### Concentration and coordination of expert opinions

Calculate the mean ($\overline{x}$) of all indicators after assignment by the 5-Likert scale. The higher the score, the higher the importance of the indicator. It is considered to be of high importance with a mean score ≥ 3.5 for each indicator. After the first round of questionnaires, no experts proposed to add a new indicator. The results of the indicators are shown in Table 2. Among them, the arterial puncture hematoma, hyperlipidemia, gender, marital status, and hospitalization for other diseases were less than 3.5 points. A high concentration is indicated by a low value of the CV, and the ideal is CV ≤ 0.35 [10]. The CV of the marital status and gender in this round is higher than 0.35. So, we excluded the above five indicators that did not meet the criteria for inclusion in the study and included other indicators in the second round of the questionnaire. The Kendall coefficient of concordance represents the consistency of all the experts in the study on the indicators, with *p* < 0.05 being statistically significant [11]. The Kendall coefficient of concordance for the first round of study was 0.19 (*p* < 0.001) (Table 3). In the second round of investigation, the members of the expert group generally believed that the current round of indicators had high reliability and feasibility, and did not propose any additions and deletions of indicators. Therefore, after two rounds of modified Delphi method, we obtained the final indicator system. The second round of research Kendall coefficient of concordance was 0.15 (*p* < 0.001), which indicates that all the experts reached a high consistency.

**Table 2:** Delphi method results

Indicators	Mean	Standard deviation (SD)	Median (P25, P75)	CV
Aneurysm treatment	4.56	0.86	5 (4.5, 5)	0.19
Intraoperative rupture	4.78	0.65	5 (5, 5)	0.13
Residual neck	3.83	1.10	4 (3, 5)	0.29
Aneurysm recurrence	3.94	1.11	4 (3, 5)	0.28
Rupture during follow-up	5.00	0.00	5 (5, 5)	0.00
Treatment-related complications	4.67	0.77	5 (5, 5)	0.16
Cerebral infarction	4.61	0.78	5 (4.75, 5)	0.17
Arterial puncture hematoma^a^	3.11	0.96	3 (2, 4)	0.31
Intervention materials	4.06	0.80	4 (3, 5)	0.20
Simple coils	3.94	0.99	4 (3, 5)	0.25
Stent or balloon-assisted coils	4.00	0.77	4 (3, 5)	0.19
Stent or flow diverter	3.83	0.79	4 (3, 4.25)	0.20
Drug complications	4.00	0.91	4 (3, 5)	0.23
Antiplatelet drug	3.83	1.10	3 (3, 5)	0.29
Anticoagulant drug	3.56	0.98	3 (3, 4.25)	0.28
Aneurysm characteristics	4.06	0.94	4 (3, 5)	0.23
Size	4.33	0.84	5 (3.75, 5)	0.19
Number	3.89	0.96	3.5 (3, 5)	0.25
Location	4.00	0.91	4 (3, 5)	0.23
Economic status	3.72	0.96	3 (3, 5)	0.26
Treatment costs	3.89	0.96	3.5 (3, 5)	0.25
Proportion of treatment costs to household income	4.11	0.96	4.5 (3, 5)	0.23
Source of expenses	4.33	0.91	5 (3, 5)	0.21
Life history	3.50	0.71	3 (3, 4)	0.20
Smoke	3.89	1.02	4 (3, 5)	0.27
Drinking	3.72	1.01	4 (3, 4)	0.27
Sleep time	3.5	1.10	4 (2, 4)	0.31
Physical exercise	4.00	0.91	4 (3, 5)	0.23
Past history	3.56	0.70	3.5 (3, 4)	0.19
Hypertension	4.28	0.89	5 (3, 5)	0.21
Diabetes	4.00	0.97	4 (3, 5)	0.24
Hyperlipidemia^a^	3.44	0.98	3 (3, 4)	0.29
Heart disease	3.78	0.94	3 (3, 5)	0.25
Mental and psychological factors	4.33	0.77	4.5 (4, 5)	0.18
Anxiety	4.11	0.90	4 (3, 5)	0.22
Depression	4.06	1.06	4.5 (3, 5)	0.26
Demographic information	3.56	0.51	4 (3, 4)	0.14
Gender^a^	3.17	1.20	3.5 (2.75, 4)	0.38
Age	3.72	0.96	3.5 (3, 5)	0.26
Education level	3.78	1.11	4 (3, 5)	0.29
Marital status^a^	2.78	1.17	3 (2, 4)	0.42
Work ability recovery	4.28	1.18	5 (3, 5)	0.28
mRS change	4.33	0.97	5 (3, 5)	0.22
Diagnosis to treatment time	3.83	0.99	3 (3, 5)	0.26
Follow-up time	3.61	0.78	3 (3, 4)	0.22
Other system complications	4.11	1.08	5 (3, 5)	0.26
Hospitalization for other diseases^a^	3.11	0.67	3 (3, 3)	0.22
Family history of cerebrovascular disease	3.72	0.89	3 (3, 5)	0.24
History of cerebrovascular disease	4.06	0.99	4.5 (3, 5)	0.25

^a^$$ \overline{x} $$ < 3.5 or CV > 0.35

**Table 3:** Kendall coefficients of concordance for two rounds of expert consultation

Factors	Kendall coefficients of concordance	*x*^2^	*p* value
First round	0.19	167.2	< 0.001
Second round	0.15	118.9	< 0.001

### The influencing factors screened by modified Delphi method

As shown in Table 2, indicators screened by the modified Delphi method include aneurysm treatment (intraoperative rupture, residual neck, aneurysm recurrence, rupture during follow-up), treatment-related complications (cerebral infarction), intervention materials (simple coils, stent or balloon-assisted coils, stent or flow diverter), aneurysm characteristics (size, number, location), drug complications (antiplatelet drug, anticoagulant drug), economic status (treatment costs, proportion of treatment costs to household income, source of expenses), life history (smoke, drinking, sleep time, physical exercise), past history (hypertension, diabetes, heart disease), mental and psychological factors (anxiety, depression), demographic information (age, educational level, work ability recovery), mRS change, diagnosis to treatment time, follow-up time, other system complications, family history of cerebrovascular disease, and history of cerebrovascular disease.

## Discussion

With the advancement of radiographic technology, the detection rate of UIA patients has increased significantly [1, 2]. Compared with the general population, the HRQoL level of the UIA patient was significantly lower due to concerns about aneurysm disease, fear of aneurysm rupture, and disease-related symptoms [12]. In recent years, endovascular treatment of UIAs has become a main strategy as result of the superiority over microsurgery clipping in both morbidity and mortality [13, 14]. Studies reported that the long-term HRQoL levels in patients with UIAs who underwent endovascular treatment have been improved compared with the preoperative [12, 15]. However, even in patients with asymptomatic UIAs, long-term postoperative HRQoL is still significantly lower than the general population [3, 16]. Many UIA patients have not been able to work and study normally after long-term recovery, which greatly affects the quality of life for these patients, considering the high prevalence and the increasing rate of radiographic detection of UIAs in the Chinese population. In addition, with the progress of society and economy, the requirements for quality of life and work ability for UIA patients also increased. Recognizing the underlying factors of poor HRQoL in UIA patients who underwent endovascular treatment and implementing further intervention and guidance from clinical management and daily life for them are essential.

In this research, a modified Delphi method was used to analyze the potential factors affecting postoperative HRQoL recovery in patients with UIAs. We found that in terms of aneurysm treatment, indicators such as treatment outcomes, choice of intervention materials, and treatment-related complications were considered to be important influencing factors. Keeping a healthy lifestyle is vital for UIA patients. Active and effective management of comorbidities such as heart disease, hypertension, and diabetes is essential to improve HRQoL for these patients. The level of education is considered to be an important influencing factor for UIA patients, which may be related to a clear understanding of the aneurysm disease among highly educated people, thus avoiding the excessive psychological burden. The diagnosis to treatment and follow-up time may also lead to differences in HRQoL.

There are certain limitations of the modified Delphi method. It is necessary to use a large sample of clinical cases and multicenter study to analyze the influencing factors screened by the modified Delphi method to obtain more accurate research results. Whatever, it is undeniable that the modified Delphi method has important guiding value for the screening and confirmation of indicators that have not yet reached a consensus.

## Conclusion

In this study, the factors affecting the recovery of HRQoL after endovascular treatment in patients with UIAs were analyzed and confirmed by the modified Delphi method, which provided a valuable evidence for the clinical management and daily life guidance for UIA patients.

## Data Availability

The datasets used and/or analyzed during the current study are available from the corresponding author on reasonable request.

## Abbreviations

Ca: Judgment basis of the indicators
Cr: Authoritative coefficient
Cs: Familiarity with the indicators
CV: Coefficient of variation
HRQoL: Health-related quality of life
IA: Intracranial aneurysm
ICH: Intracerebral hemorrhage
IVH: Intraventricular hemorrhage
mRS: Modified Rankin Scale
SAH: Subarachnoid hemorrhage
UIA: Unruptured intracranial aneurysm
